# Seasonal fluctuations in fitness result in severe reductions in effective population size

**DOI:** 10.1093/g3journal/jkag252

**Published:** 2026-09-03

**Authors:** Olivia L Johnson, Raymond Tobler, Joshua M Schmidt, Christian D Huber

**Affiliations:** Department of Biology, The Pennsylvania State University, University Park, PA 16802, United States; School of Biological Sciences, University of Adelaide, Adelaide, SA 5000, Australia; Evolution of Cultural Diversity Initiative, The Australian National University, Canberra, ACT 2600, Australia; Department of Ophthalmology, College of Medicine and Public Health, Flinders University, Bedford Park, SA 5052, Australia; Department of Biology, The Pennsylvania State University, University Park, PA 16802, United States

**Keywords:** fluctuating selection, effective population size, seasonal fluctuations, *Drosophila melanogaster*

## Abstract

Genetic evidence for fluctuating selection has begun to accumulate for different species over the past few decades, especially for the *Drosophila* genus, where studies have reported hundreds of loci undergoing putatively adaptive oscillations across successive seasons. However, most theoretical and simulation studies of fluctuating selection have relied on abstract or weakly parameterized models, making it difficult to assess their relevance for natural populations. In this study, we simulate multilocus seasonally fluctuating selection acting on standing genetic variation under a recently developed model and examine its effect on the variance effective population size (*N*_e_) at a genome-wide scale. By recapitulating genomic, demographic, and evolutionary parameters from natural *Drosophila* populations in our simulations, we were able to reproduce allele frequency oscillations reported in recent studies and show that these lead to ∼50% genome-wide reductions in *N*_e_. We also demonstrate that *N*_e_ reductions are well predicted by the maximum frequency amplitude among all adaptively fluctuating loci, and that the frequency amplitudes are largely determined by the number of adaptively fluctuating loci and the strength of their epistatic interactions. Our results demonstrate that fluctuating selection can substantially reduce effective population size and underscore the importance of temporally variable selection in shaping genome-wide patterns of variation beyond classical models.

## Introduction

Effective population size is a central parameter in population genetics, governing the strength of genetic drift and shaping patterns of genetic variation. Although effective population size is often interpreted in terms of demographic processes, it can also be strongly influenced by selection through its effects on the distribution of reproductive success among individuals. Different formulations of *N*_e_ capture these dynamics from distinct perspectives, including variance effective size, which reflects fluctuations in reproductive success across generations, and coalescent effective size, which is inferred from levels of genetic diversity (Crow 1954; Sjödin et al. 2005; Wakeley and Sargsyan 2009; Wang et al. 2016; Waples 2022).

Selection is known to systematically alter these measures. Positive selection and purifying selection can reduce effective population size by increasing variance in reproductive success and by depleting linked neutral diversity through selective sweeps and background selection (Charlesworth 2012; Comeron 2014, 2017; Corbett-Detig et al. 2015; Buffalo 2021; Charlesworth and Jensen 2022; Achaz and Schertzer 2023). In contrast, balancing selection can maintain genetic variation and thereby increase estimates of effective size (Charlesworth 2006; Rodríguez de Cara et al. 2023).

One selective process that has received increased attention recently but remains relatively unexplored in relation to effective population size is fluctuating selection—i.e. adaptive scenarios where selection changes in direction or intensity across time (Dobzhansky 1947; Haldane and Jayakar 1963; Gillespie 1991; Johnson et al. 2023). A number of theoretical and empirical studies have recently demonstrated that fluctuating selection can have wide-ranging effects on levels of population genetic diversity (Barton 2000; Taylor 2013; Huang et al. 2014; Wittmann et al. 2023). In particular, a recent theoretical study reported a 30% reduction in genome-wide diversity when seasonal fluctuating selection acted upon a single site, with these impacts also extending across unlinked neutral sites on different chromosomes (Wittmann et al. 2023). This remarkable genome-wide reduction in diversity was caused by the onset of serial reproductive bottlenecks occurring at the start of each season, where the least fit individuals from the previous season exhibited a significant reproductive advantage in the present season, resulting in greatly increased variability in offspring number between individuals (Wittmann et al. 2023).

These results indicate that species susceptible to temporally fluctuating selection pressures may also be expected to experience a severe decrement in effective population size. Moreover, while our understanding of the scope and impact of fluctuating selection in natural populations is currently limited to studies conducted on a handful of species (Kelly 2022; Pfenninger and Foucault 2022; Johnson et al. 2023; Lynch et al. 2024), research on natural and experimental outdoor populations of *Drosophila* suggest that hundreds to potentially thousands of fluctuating loci are under seasonal selection in these cosmopolitan taxa (Bergland et al. 2014; Behrman et al. 2018; Machado et al. 2021; Rudman et al. 2022; Bitter et al. 2024; Nunez et al. 2024). However, the impact of this pervasive fluctuating selection signal on effective population size remains unknown, and our ignorance further extends to how factors like the number of fluctuating loci, selection strength, and census population size combine to mediate impacts on effective size. In the present study, we employ forward simulations that draw upon a recently published multilocus model of seasonal adaptation (Wittmann et al. 2017) and parameter ranges informed by empirical studies of *Drosophila melanogaster* (Bergland et al. 2014; Machado et al. 2021; Rudman et al. 2022; Bitter et al. 2024) to provide new insights into the impact of fluctuating selection on instantaneous variance effective population size (*N*_e_) under plausible evolutionary scenarios.

## Methods

### Parameter selection for the segregation lift model

The segregation lift (SL) model consists of 2 parts: a score of the contribution of the loci in a genotype (*z*_s/w_; see equation 1 and Table 1) and a fitness equation (see equation 2) (Wittmann et al. 2017). For the score component, each seasonal locus was allocated seasonal dominance coefficients (*d*_s/w_) and effect sizes (Δ_s/w_). Dominance coefficients for each season were drawn independently from a uniform distribution with a minimum of 0 and a maximum of 1, with only seasonal loci having mean dominance values greater than 0.5 being retained because these loci are more likely to become stable polymorphisms (Wittmann et al. 2017). Effect sizes for each seasonal locus were drawn from a bivariate normal distribution with a mean of 0, a standard deviation of 1, and a correlation coefficient of 0.9, with both values being exponentiated to create a log-normal distribution (Wittmann et al. 2017). Correlated values were used since smaller differences between the summer and winter effect sizes are more likely to produce stable polymorphisms (Wittmann et al. 2017). The contribution of a locus was either the seasonal effect size, if homozygous for the seasonally favored allele, the product of the seasonal effect size and the dominance coefficient if heterozygous, or 0 if homozygous for the unfavored allele (Table 1). The seasonal score is calculated for each individual in each generation as the sum of each locus’s contribution depending on the individual's genotype (equation 1). This score is then used to calculate each individual's fitness as per equation 2. Epistasis levels recreate a diminishing-returns fitness model (*y* range: 0.5, 1, 2, 4, 8, 12, 16, 20), as evidence for this model has appeared in several empirical studies (Chou et al. 2011; Khan et al. 2011; Kryazhimskiy et al. 2014) and theoretical studies show that it is more likely to produce stable fluctuating loci that experience reasonable reversals of dominance (whereas other forms of epistasis require almost complete reversal of dominance) (Wittmann et al. 2017). Finally, we followed empirical reports of the number of segregating seasonal loci in natural *Drosophila* populations (Rudman et al. 2022; Bitter et al. 2024) and introduced either 100, 200, or 500 seasonal loci (*l*) in our initial simulations. Importantly, our selection model assumes “soft” selection, i.e. selection does not affect the census population size, and the *N*_e_ reduction is solely attributable to the increased variance in offspring number.


(1) $$z_{s/w}=\;\overset{L}{\underset{l=1}{\sum }}\;C_{l}$$

(2) $$w(z)=(1+z{)}^{y}$$

**Table 1.** Contribution of seasonal loci (*C_l_*) to fitness.

Season	*C_l_*_,WW_	*C_l_*_,SW_	*C_l_*_,SS_
Winter	Δ_w_	Δ_w_*d*_w_	0
Summer	0	Δ_s_*d*_s_	Δ_s_

The contribution of a seasonal locus is conditioned on the genotype at that locus and the current season. If the locus is homozygous for the seasonal allele, the contribution is the effect size of the seasonal allele. If the locus is homozygous for the allele of the opposing season, the contribution is 0. If the locus is heterozygous, the contribution is the product of the effect size and the dominance coefficient for that given season.

### Forward simulations of fluctuating allele frequency trajectories

All simulations were performed using *SLiM* (v 4.0.1; Haller and Messer 2023), starting with simulations designed to quantify the allele frequency trajectories of unlinked seasonal loci under the SL model. Simulations were performed across combinations of the initial number of seasonal loci and epistasis intensity (*y*: 0.5, 1, 2, 4, 8, 12, 16, and 20; *l*: 100, 200, and 500). To improve computational feasibility, the main simulations used a census population size of 500,000 diploid individuals, 5 generations per seasonal environment, and a total duration of 18,000 generations. Relative to a reference parameterization of 1 million individuals and 10 generations per seasonal environment, census population size and seasonal duration were reduced by a factor of 2, while the per-generation recombination rate was increased by a factor of 2. This approximately preserves the expected amount of recombination per seasonal interval.

Selection strength was not rescaled because fitness in the segregation-lift model is determined by a nonlinear multilocus score rather than by an independent selection coefficient at each locus. The scaled simulations therefore should not be interpreted as exactly preserving all population-scaled parameters or the precise balance between selection and drift in the reference population. Instead, they provide a computationally tractable approximation whose resulting numbers of segregating loci and allele-frequency amplitudes can be compared with empirical estimates from natural *Drosophila* populations. Due to computational constraints, simulations beginning with 500 seasonal loci were limited to epistasis intensities of 8, 12, and 20, as preliminary simulations indicated that this range produced allele-frequency dynamics most consistent with the empirical observations considered here.

A second set of allele-frequency simulations used a smaller population size of 10,000 diploid individuals to provide greater temporal resolution of the initial allele-frequency dynamics. All other parameter ranges were unchanged. Because this reduction in population size was not accompanied by a corresponding rescaling of the fitness model, these simulations were used only for qualitative examination of early allele-frequency trajectories and were not interpreted as quantitatively equivalent to the larger-population simulations.

### Multiple linear regression of allele frequency amplitude for estimating selection parameters

We used the information obtained in the allele frequency simulations to develop a quantitative model of epistasis intensity (*y*) as a function of the number of seasonal loci and the amplitude of seasonal allele frequency change. We alternatively used the mean, median, and 90th percentile of seasonal loci frequency changes as one of the independent variables, with the mean amplitude giving the best model fit overall (*r*^2^ = 0.988; cf. median: *r*^2^ = 0.963, 90th percentile: *r*^2^ = 0.985). An ANOVA model comparison suggested that adding a 2-way interaction term significantly improved the model fit (*P* < 0.001; *r*^2^ = 0.989; see equation 3 and Supplementary Table 2, where *y* is the epistasis value, *n* is the final number of segregating seasonal loci, and *x* is the mean amplitude of seasonal allele frequency fluctuation) and this model was subsequently used to predict epistasis (*y*).


(3) log(y)∼−0.153+1.310log(n)+0.766log(x)+0.180(log(n)×log(x))

A multiple regression model was also used to determine the number of initially segregating loci required to obtain the final number of segregating loci after seasonal allele frequency oscillations had stabilized. This model predicts the required initial number of segregating loci (*l*) from the epistasis parameter (*y*) and the final number of segregating loci (*n*) (Supplementary Fig. 12). Again, adding an interaction term to the model (equation 4) significantly improved the fit (*P* < 0.001).


(4) l∼4.407+1.154n+0.443y+0.009(n*y)

Using the 2 fitted regression models shown in equations 3 and 4, we predicted the epistasis intensities and the initial number of segregating loci needed to replicate the mean amplitude of allele frequency fluctuation and the number of segregating loci reported in studies of natural *D. melanogaster* populations (Table 2). For the final number of segregating seasonal loci, we took values reported in recently published outdoor *D. melanogaster* cage experiments (Rudman et al. 2022; Bitter et al. 2024), since the authors used linkage disequilibrium, hitchhiking, and parallel allele frequency changes to provide a conservative estimate of the number of independent seasonal loci coming under selection. For mean frequency amplitude, we used a range of values obtained from natural and experimental *D. melanogaster* studies (Bergland et al. 2014; Machado et al. 2021; Rudman et al. 2022; Bitter et al. 2024), utilizing criteria that are summarized in Table 2.

**Table 2.** Parameters for genome-wide simulations.

Number of causal loci	Mean amplitude	References	Derived *y*	Derived initial loci
111	0.05	The number of genes that overlapped with clusters in Rudman et al. (2022)	11	148
90	0.08	The maximum number of clusters identified at any one time point in Rudman et al. (2022)	15	127
264	0.02	The number of seasonal loci suggested to underlie the signal identified in Bitter et al. (2024)	9.5	335
27	0.04	The number of clusters that contained SNPs linked to SNPs that overlapped. These were then used to form the 9 superclusters from Rudman et al. (2022)	2	37
9	0.35	Minimum number of causal loci having formed 9 superclusters from seasonal SNPs in Rudman et al. (2022). The average amplitude of the top 9 SNPs in each study of natural populations with available data was found to be ∼ 35% (Bergland et al. 2015; Machado et al. 2019)	4.5	18

Empirical estimates of number of causal fluctuating loci and the mean amplitude of fluctuation used to derive the epistasis parameter (*y*) and number of initial loci used in our empirically informed simulations of natural Drosophila populations. The rationale and references for the empirical values are included for each combination of values.

### Empirically informed simulations of natural Drosophila populations

We used the empirically derived parameters estimated from our multiple linear regression models to simulate fluctuating selection under the SL model in diploid individuals that each carried both *Drosophila* autosomes, with chromosome lengths taken from the FlyBase (Jenkins et al. 2022) and recombination rates following Comeron et al. (2012). At the start of the simulations, seasonal alleles were introduced at 50% frequency at random positions across 3 of the 4 chromosome arms, leaving the final chromosomal arm (3R) with no seasonal loci to facilitate recording of neutral allele frequency changes on a genomic region unlinked to selected loci. We expect the mutations to be in linkage equilibrium by introducing them in this manner. Simulations used to estimate variance effective population size employed a constant census population size of 500,000 diploid individuals, 5 generations in each of the 2 seasonal environments, and a total duration of 10,000 generations. As in the allele-frequency simulations described above, population size and the number of generations per seasonal environment were reduced by a factor of 2 relative to the reference parameterization, while the per-generation recombination rate was increased by a factor of 2. This scaling approximately preserves the expected amount of recombination per seasonal interval but does not guarantee exact preservation of the balance between selection and drift.

We also conducted simulations representing boom-bust demography. In these simulations, the winter census population size was reduced to 50,000 diploid individuals, compared with a summer census population size of 500,000. These values correspond to reference population sizes of 100,000 and 1 million individuals, respectively, before the 2-fold population-size reduction used for computational tractability.

### Effective population size estimation

To measure the reduction in *N*_e_ caused by fluctuating selection in the empirically informed *Drosophila* simulations, neutral mutations starting at 50% frequency were inserted every 100 kb across the simulated genomes at multiple sampling time points, and the standardized variance, *F*, was calculated for each neutral mutation after *g* generations of change, according to equation 5 (Waples 1989).


(5) $$F=\frac{{\left(\mathrm{allele}\;\mathrm{frequency}\;\mathrm{change}\right)}^{2}}{0.25}$$

These *F* values were averaged separately across chromosome arms with and without seasonal loci (i.e. 2L, 2R, 3L, or 3R) and used in equation 6 to calculate the *N*_e_, with *g* signifying the number of generations (i.e. 10 in this case) (Waples 1989; Jónás et al. 2016).


(6) $$N_{e}=\frac{-g}{2\;\ln(1-\;F)}$$

Neutral mutations were removed from the simulation following the estimation of *N*_e_ and reintroduced at 50% at the next sampling point. Neutral alleles were introduced at 50% frequency as standing neutral markers to maximize precision when estimating temporal allele-frequency variance. These alleles are not intended to represent newly arising mutations, but rather provide a standardized set of neutral loci for estimating instantaneous variance effective population size over short sampling intervals.

To capture *N*_e_ changes across successive generations of the seasonal cycle, we ran a separate set of simulations for 2,500 generations (i.e. 5,000 unscaled), and we inserted neutral mutations starting at 50% frequency in each of the next 10 simulated generations (i.e. a single seasonal cycle, successively removing alleles from the previous generation) and measured *N*_e_ using equation 6 (where *g* = 1). To examine if the discrepancy observed between *N*_e_ calculated over 1 or 10 simulated generations was an artifact (Waples 2016, 2022), we repeated this process but let each neutral locus segregate for 30 generations and calculated *N*_e_ at each generation (i.e. *g* = 1, 2, 3, …, 30), limiting calculations to loci that were still segregating after 3 complete seasonal cycles.

### Estimating reduction in *N*_e_ from maximum allele frequency amplitudes

We used standard linear regression to quantify the relationship between maximum allele frequency amplitude and the reduction in *N*_e_, obtaining the model shown in equation 7.


(7) Reduction∼0.056−1.209(Maximumamplitude)

We used this equation to estimate the reduced *N*_e_ in natural *Drosophila* populations previously reported to be under fluctuating selection (Bergland et al. 2015; Machado et al. 2019), obtaining maximum allele frequencies from data retrieved from the relevant Dryad repositories (code available at Johnson et al. 2026).

### Offspring capping

Our simulation of strong seasonal selection can produce an unrealistically large number of offspring for a small number of highly fit parental individuals. We capped the number of offspring at 10 simulated individuals (i.e. 20 individuals prior to parameter downscaling), using *SLiM*'s “modifyChild” callback. This allowed alternate parents to be allocated to individuals of a new generation if the ones originally picked had already contributed to 10 individuals.

### Exploratory analysis of an asymmetric seasonal regime

We simulated an asymmetric seasonal regime with 8 scaled generations in one selective environment and 2 in the other, corresponding to 16 and 4 unscaled generations, respectively. This preserved a total of 20 generations per annual cycle while introducing a strong asymmetry in seasonal duration. We examined 3 parameter combinations (*l* = 127, 37, and 18; *y* = 15, 2, and 4.5, respectively), with 10 replicate simulations per combination. We estimated variance *N*_e_ for each replicate and compared the results with those obtained under the corresponding symmetrical seasonal regime.

## Results

### Simulating adaptive seasonally fluctuating loci

To assess the impact of fluctuating selection on *N*_e_, we used the forward population genetic simulator *SLiM* (v 4.0.1; Haller and Messer 2023) to replicate multilocus adaptation across 2 alternating seasonal environments (i.e. summer and winter; s/w) under the SL model developed by Wittmann and colleagues (Wittmann et al. 2017). The SL model comprises a dynamic fitness landscape where the seasonal scores for each individual (i.e. *z*_s_ for summer and *z*_w_ for winter; see equation 1) are obtained by summing the contributions of all seasonally adaptive loci (each locus-specific contribution, *C_l_*, being the product of a seasonal dominance coefficient (*d*_s/w_) and effect size (Δ_s/w_); Table 1; see Methods). This score is converted to fitness, *w*(*z*), via a power transformation (scale = *y*; equation 2) that allows for nonadditive contributions across seasonal loci (i.e. epistasis; also see Methods for further discussion).

For our initial simulations, we focused on quantifying the dynamics of allele frequency fluctuations of seasonally adaptive loci (henceforth seasonal loci) under a simple demography where population size remained constant at 1 million individuals across 36,000 generations and each season had 10 generations. Note that we were able to improve the computational efficiency of our simulations while still obtaining representative results by downscaling each of these parameters by a factor of 2 (see Methods), but we refer to the unscaled parameter values throughout for convenience.

Initially, we sought to identify the impact of the number of seasonal loci and epistasis intensity on the magnitude of frequency fluctuations and the number of generations needed to achieve stable oscillations. Accordingly, separate simulations were run using different combinations of both variables—seasonal locus count ranging between 100 and 500 and epistasis intensity (*y*) ranging from 0.5 to 20; ranges based on reports from relevant *D. melanogaster* studies (Bergland et al. 2014; Machado et al. 2021; Rudman et al. 2022; Bitter et al. 2024)—with all loci being introduced in the first generation with each seasonal allele at 50% frequency. Seasonal dominance and effect sizes were randomly drawn from independent distributions (see Methods), facilitating a broad range of seasonal allele frequency fluctuations across all seasonal loci. The fitness function (equation 2) induces diminishing-returns fitness scenarios for all epistasis intensities, as this type of epistasis has prior support from empirical studies (Chou et al. 2011; Khan et al. 2011; Kryazhimskiy et al. 2014; Wittmann et al. 2017) and produces stable fluctuations with plausible properties (Wittmann et al. 2017) (see Methods). After running each parameter combination 20 times, we evaluated trends in allele frequency changes for all seasonal loci sampled over 3 consecutive seasonal cycles at the commencement, midpoint (i.e. from 18,000 generations), and end of the simulation (i.e. from 36,000 generations).

### Fluctuating allele frequencies depend on the number of segregating seasonal loci and epistasis intensity

Across all simulations, we observe a continuous decline in the number of segregating loci across time (Supplementary Fig. 1), with loci with similar seasonal effect size (i.e. Δ_s_/Δ_w_ ≅ 1) being more likely to remain segregating throughout, while those with skewed effects (i.e. Δ_s_/Δ_w_ ≪ 1 or Δ_s_/Δ_w_ ≫ 1) were more prone to loss (Supplementary Fig. 2), corroborating theoretical results (Wittmann et al. 2017). The rate of seasonal locus loss is strongly dependent on the epistasis intensity, with weakly epistatic loci becoming lost at a steady rate, whereas loci experiencing stronger epistasis are lost at higher rates initially, before leveling off toward the end of the simulation.

Despite the continual loss of seasonal loci through time, seasonal allele frequency fluctuations had stabilized by 18,000 generations, with mean allele frequency amplitudes (i.e. seasonal frequency range averaged over successive cycles at each sampling point) ranging between ∼0.4% and 16.5% (see Methods; Supplementary Fig. 3). Notably, the relationship between seasonal locus loss rate and epistasis was maintained after downscaling the population size by an order of magnitude (Supplementary Fig. 4; see Methods), with increased sampling during the early stages of the downscaled simulations revealing that the seasonal allele frequency amplitudes had become stable by 1,200 generations across all parameter combinations (Supplementary Fig. 5). This rapid stabilization likely extends across all of our simulations, since alleles at all seasonal loci start at 50% frequency from the onset of the simulations, which is the mean value of the oscillating alleles at equilibrium, and the combined results indicate the stable frequency oscillations are maintained even as seasonal loci continue to be lost.

Strikingly, the seasonal frequency amplitudes at the end of the simulations showed a strong inverse correlation with the final number of segregating seasonal loci but a positive relationship with epistasis intensity (Fig. 1). After log-transforming each variable, we were able to fit a linear regression model to our simulated data that accurately predicted the mean seasonal frequency from linear combinations of seasonal loci number and epistasis intensity (*r*^2^ = 0.988). Similar fits were obtained for regression models predicting the median (*r*^2^ = 0.963), maximum (*r*^2^ = 0.921), and 90th percentile frequency amplitudes (*r*^2^ = 0.985), indicating that frequency fluctuations in the center and upper tail of the distribution are highly predictable. Further inspection of the downscaled simulations showed that this linear relationship between the log-transformed oscillating allele frequencies and segregating seasonal loci had converged by 18,000 generations for all epistasis intensities, with convergence being faster for loci with higher epistasis intensities (Supplementary Fig. 6).

**Fig. 1.**
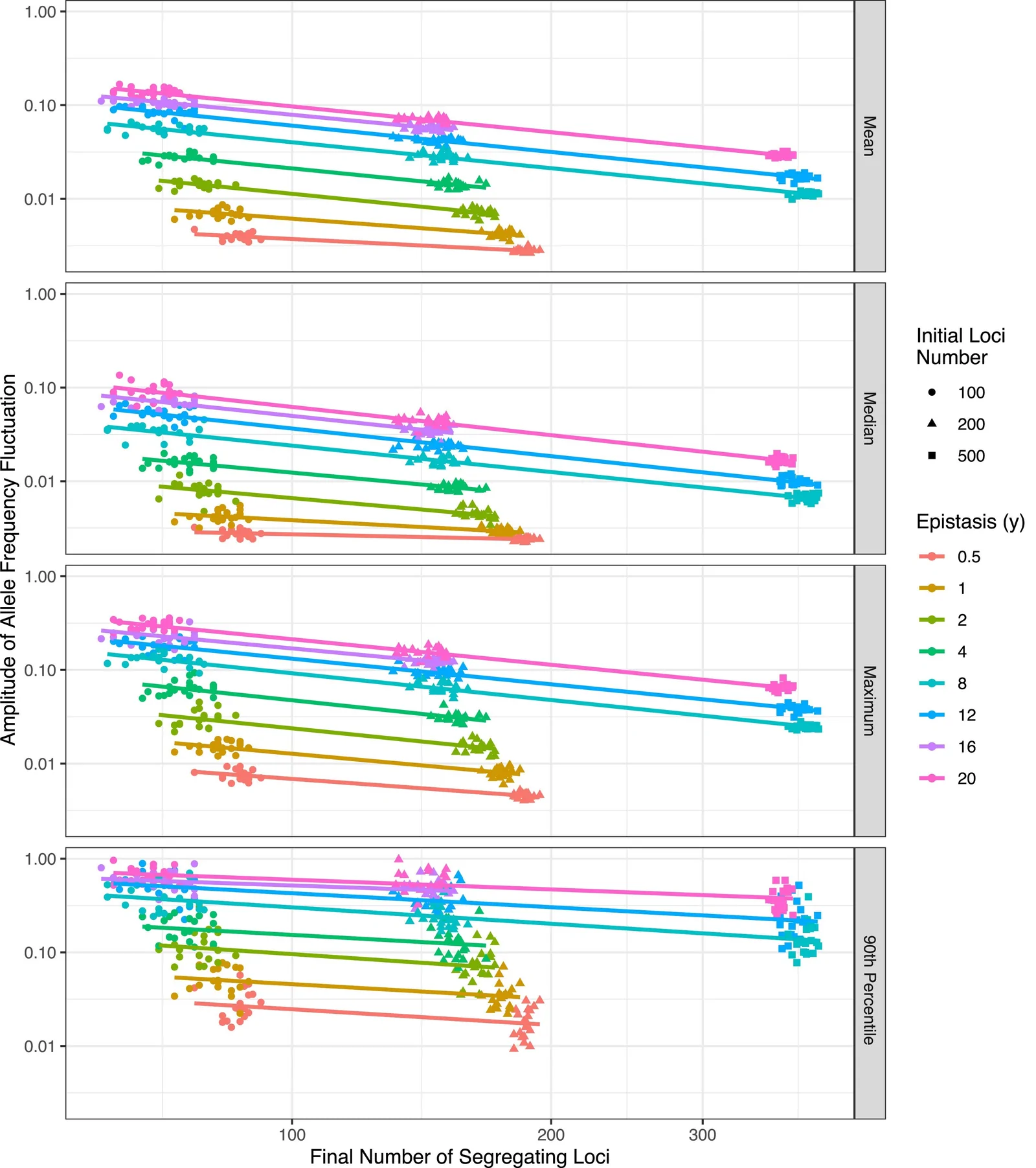
Relationship between number of segregating loci, epistasis, and amplitude of seasonal allele frequency fluctuation. Each panel depicts the relationship between the initial and final number of segregating loci (sampled at 18,000 generations) and the mean, median, maximum, or 90th percentile of allele frequency fluctuations, for different epistasis values. The amplitude of the seasonal fluctuation is shown on the *y*-axis. The initial loci number is visualized by the shape of the points, with each point representing a single simulated replicate (of which there are 20 for each initial loci number and epistasis value). The color of the points and lines indicated the epistasis value.

These relationships prompted us to fit regression models to the simulated datasets that predict the expected epistasis intensity as a linear function of the number of segregating seasonal loci and their mean oscillation magnitude (see equations 3 and 4 in Methods). Because the number of segregating loci was also predictable from the number of starting loci (Fig. 1), we used the best-fitting regression model to estimate the combination of epistasis intensity and initial number of seasonal loci required to replicate the number and amplitude of fluctuating loci reported in natural *D. melanogaster* populations (Table 2; see Methods). The estimated number of seasonal loci and interaction intensities were then used to parameterize simulations using the SL model, and the results were analyzed to provide the first direct estimates of *N*_e_ reductions in natural *D. melanogaster* populations experiencing seasonal fluctuating selection.

### Estimating the genome-wide effect of seasonal fluctuating selection on *N*_e_ in *D. melanogaster*

To ensure that our simulations reproduce a feasible genomic context for *D. melanogaster*, we randomly introduced a specific number of seasonal loci reported in previous studies (ranging between 9 and 264 loci; Table 2) into 1 million diploid individuals each bearing the 2 main *D. melanogaster* autosomes (with empirically informed recombination rates from Comeron et al. (2012) and seasonal loci omitted from autosomal arm 3R to facilitate *N*_e_ estimation at unlinked loci; see Methods). All simulated scenarios ran for 20,000 generations, with seasonal alleles introduced in the first generation at 50% frequency and neutral mutations introduced at the same frequency every 2,000 generations at 100 kb intervals across both chromosomes (969 in total). For each sampled time point, we recorded neutral allele frequencies across all simulated generations over a complete seasonal cycle lasting 20 generations, and used the variation in neutral allele frequencies across the seasonal cycle to measure the instantaneous variance *N*_e_ (Waples 1989; Jónás et al. 2016) (noting that generation and population size parameters were again downscaled by a factor of 2 to improve computational efficiency in the actual simulations, and neutral alleles were removed after each *N*_e_ estimation). Finally, the impact of fluctuating selection on neutral diversity levels was inferred by evaluating *N*_e_ relative to the census population size (i.e. *N*_e_/*N*_c_).

Our simulations robustly reproduced the anticipated number of segregating seasonal alleles after 20,000 generations (all but one scenario falling within the 95% confidence interval [CI], of the expected empirical values; Supplementary Table 1), though the mean seasonal frequency amplitude tended to be slightly lower than expected (reduced by 0.2% to 2% and only one scenario falling within the associated 95% CI), with the maximum discrepancy for both mean amplitude and final number of segregating loci occurring when simulating the most exaggerated seasonal fluctuations (i.e. 9 final loci with an average amplitude of 35%). These results suggest that our final *N*_e_ estimates may be slightly conservative for each of the simulated scenarios.

The impact of fluctuating selection on the genome-wide *N*_e_ was significant though highly variable, with reductions ranging between 7% and 76% relative to *N*_c_ (Fig. 2a and Supplementary 7). These reductions reached stable values within the first 2,000 generations, concordant with the rapid stabilization observed for frequency fluctuations. While the most pronounced *N*_e_ reductions occurred prior to stable frequency oscillations being reached, this pattern likely represents an artifact of the highly skewed reproductive variances resulting from all seasonal alleles commencing at 50% frequencies.

**Fig. 2.**
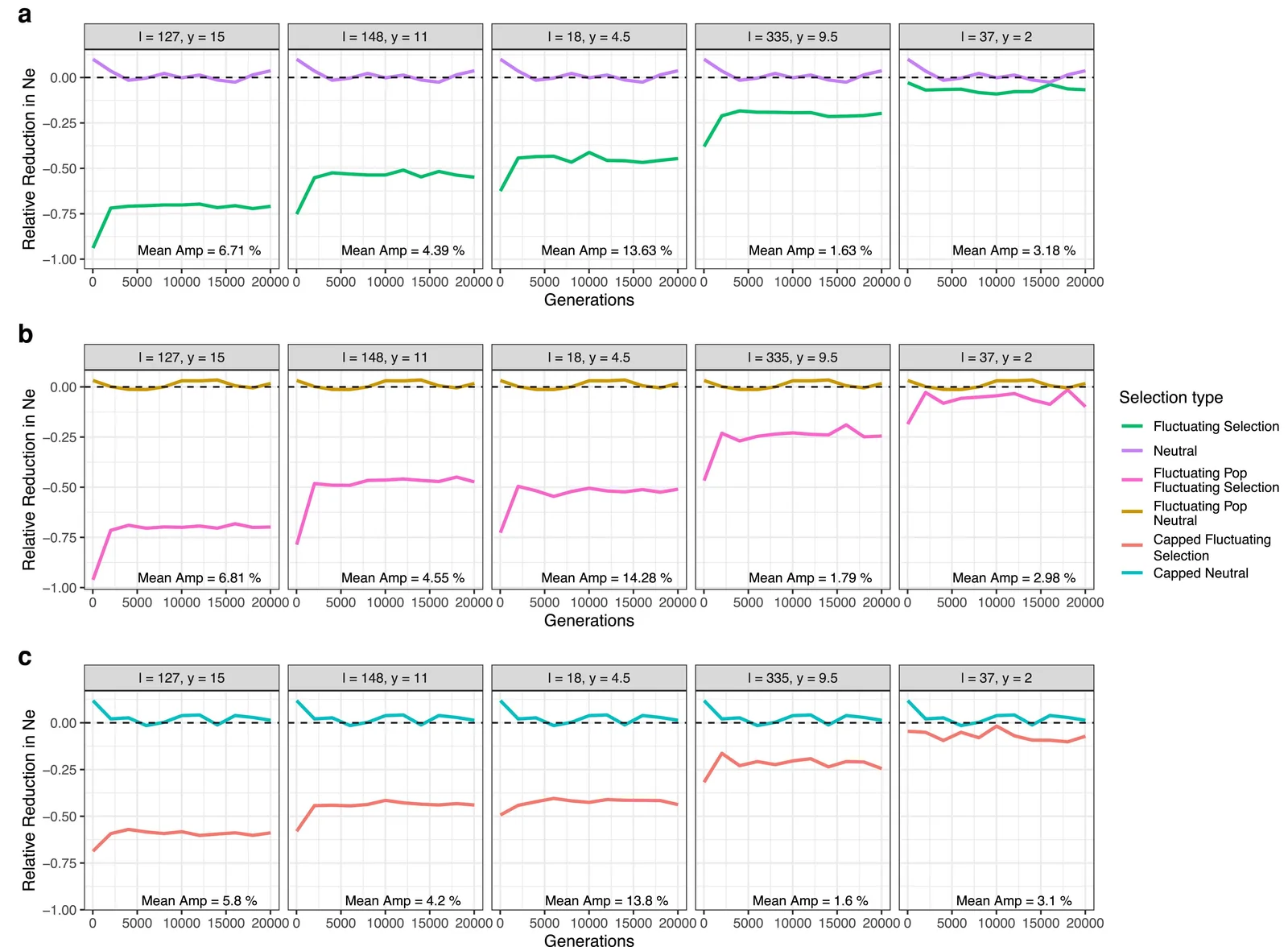
Relative reduction in effective population size due to fluctuating selection. Relative reduction in effective population size across time (generations): a) under standard conditions; b) with seasonally fluctuating population size; and c) when offspring number was capped at 20 individuals. Each panel shows effective population size for a different combination of initial loci number and epistasis value (labeled respectively in the top strip). A dashed black line depicts the neutral expectation (i.e. 0% reduction). The corresponding mean amplitude of each parameter combination is included in the bottom right of each panel.

The magnitude of *N*_e_ reduction also depended on the sampling time within seasons. We calculated *N*_e_ every generation over a seasonal cycle and observed the largest changes occurring following the seasonal transition due to increased reproductive variance over this period—though this effect is not readily discernible in scenarios where the *N*_e_ reductions were low (Fig. 3 and Supplementary 8). Notably, *N*_e_ reductions calculated across single generations within a seasonal cycle have reduced magnitudes compared to those calculated over larger timescales as *N*_e_ estimated over a single generation does not adequately capture genetic drift occurring over a full seasonal cycle (Supplementary Fig. 8). A similar downward bias has been reported for variance-based estimators of *N*_e_ when measurements are made over relatively few generations, seemingly due to the increased noise ratio in the allele frequency change estimates (Waples 2016, 2022).

**Fig. 3.**
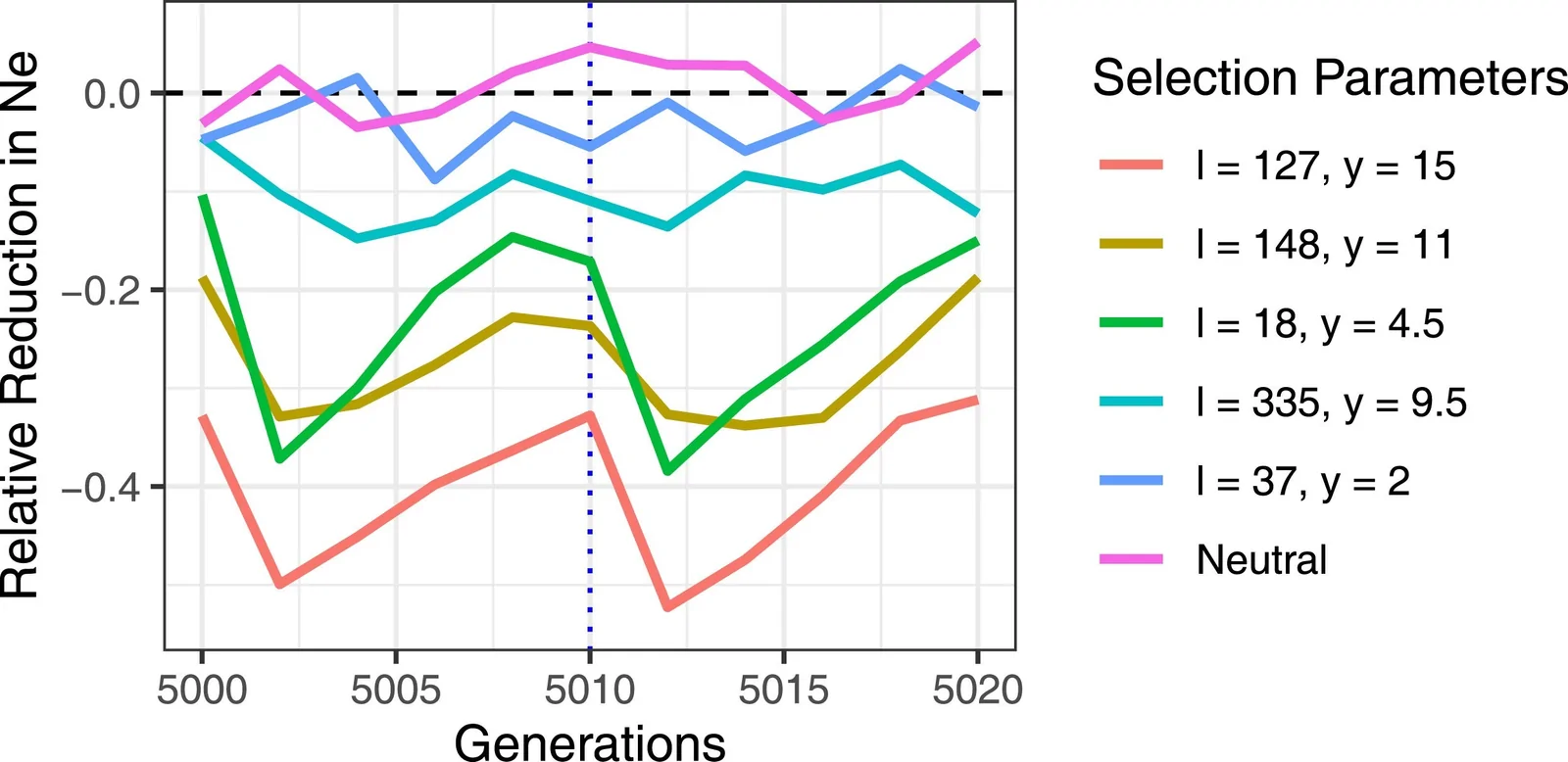
Effective population size across the seasonal cycle. Effective population size for different fluctuating selection parameters (colored lines) across the seasonal cycle, 2 seasons consisting each of 10 generations. The blue, dashed vertical line signifies the time at which the seasons change. *N*_e_ was sampled from generation 5,000 to 5,020 of the simulation as previous simulations suggested the reduction in effective population size had become stable at this time.

### Reductions in *N*_e_ scale with the largest seasonal allele frequency fluctuations

Interestingly, the greatest reduction in *N*_e_ was not observed under scenarios with the most extreme mean seasonal fluctuations, but from the scenario where ∼90 seasonal loci stably oscillated with a mean amplitude of 6.7%, which closely aligns with the mean fluctuations reported in natural and outdoor experimental *Drosophila* populations (Machado et al. 2021; Rudman et al. 2022; Bitter et al. 2024). Further, while neither the mean nor the median seasonal amplitude showed a clear relationship with *N*_e_ reduction—largely due to one scenario being an outlier (∼9 final loci, *y* = 4.5)—the maximum seasonal fluctuation displayed a strong linear correspondence with *N*_e_ reduction (*r*^2^ = 0.702). This result suggests that seasonal loci with the most extreme allele frequency fluctuations are better predictors of *N*_e_ reductions when fluctuating selection operates under an SL model.

Closer examination of the seasonal loci exhibiting the largest fluctuations showed a strong positive relationship with the mean effect size (Fig. 4a) and little dependence on the mean dominance coefficient, which instead appeared to constrain the lower limits of the frequency amplitudes (Fig. 4b). For all 5 of our modeled scenarios, the seasonal locus exhibiting the maximum frequency fluctuations tended to remain the same across time in each simulation (the only exceptions being a handful of simulations where the maximal amplitudes alternated between 2 loci). The close concordance between the maximum allele frequency fluctuation and *N*_e_ prompted us to develop another linear regression model (equation 7; Methods) where expected *N*_e_ reductions were calculated relative to the maximum seasonal frequency amplitudes. Applying this regression model to the maximum frequency amplitudes reported for natural *Drosophila* populations—i.e. 37% in Bergland and colleagues 2014 study and 45% in Machado et al.'s follow up study in 2021 (Bergland et al. 2014; Machado et al. 2021)—resulted in predicted *N*_e_ values that are ∼40% to 50% lower than the census population sizes (Fig. 5). These results are consistent with the magnitude of reductions observed from our previous regression analyses and together suggest that the continual exposure of cosmopolitan *Drosophila* species to shifting seasonal selection pressures imposes a significant constraint on the effective size of the population that has been unappreciated previously.

**Fig. 4.**
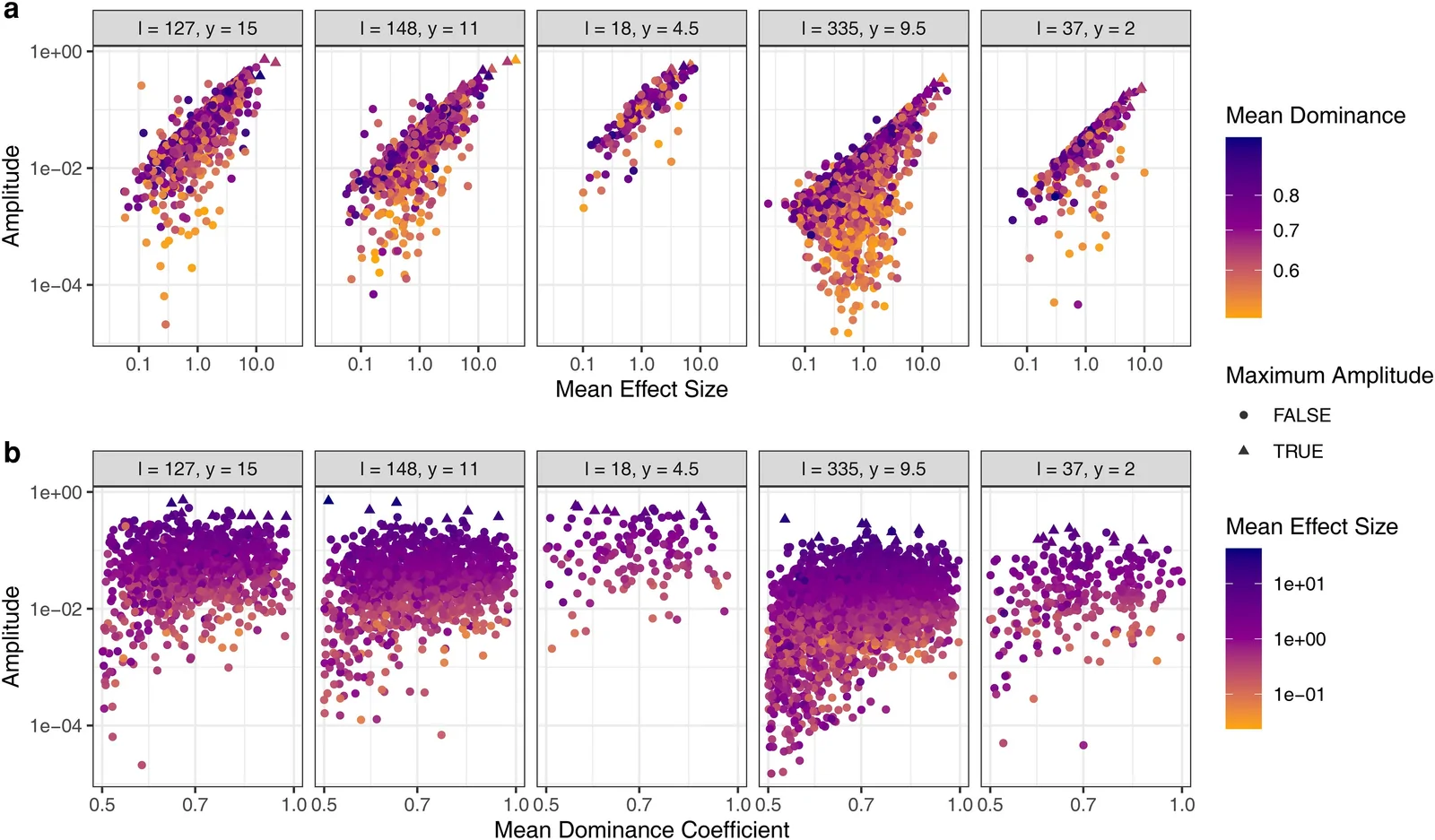
Mean effect size and mean dominance coefficient of seasonal loci segregating at the end of simulations in relation to their amplitude of fluctuation. a) The mean effect size and b) mean dominance coefficient, calculated as the average of the summer and winter values for each locus, compared to the amplitude of each locus. The colors of the points illustrate the mean dominance or effect size. Each panel depicts a different parameter combination labeled in the top strip with the initial loci number and epistasis value, respectively. Loci from all 10 simulated replicates are included in each panel. Loci identified as having the maximum amplitude at any point in a simulated replicate are depicted as triangles.

**Fig. 5.**
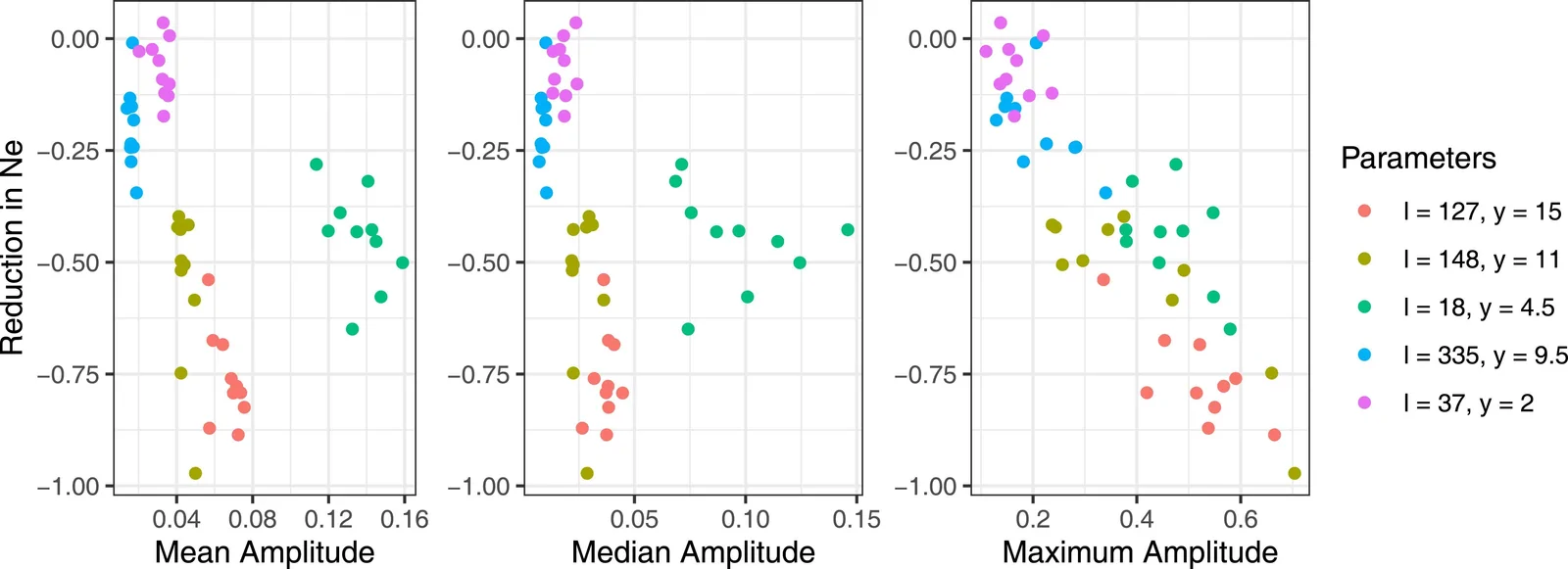
Relationship between reduction in effective population size and aspects of seasonal fluctuation (amplitude). The relative reduction in effective population size in relation to the mean, median, and maximum amplitude for each simulated replicate of each combination of initial loci number and epistasis value (colored).

### Fluctuating selection produces consistently large genome-wide *N*_e_ reductions under alternate demographic scenarios

We explored the generality of our findings under different demographic models, first simulating fluctuating selection under a wide range of constant population sizes that spanned 2 orders of magnitude (i.e. *N*_c_ ranging between 20,000 and 1 million individuals) and replicating parameters from the scenario showing the greatest reduction in *N*_e_ (75% for 127 initial loci and *y* = 15). Notably, the resulting *N*_e_ reduction did not substantially change across all 4 examined *N*_c_ values (Supplementary Fig. 9), indicating that the *N*_e_ reduction is independent of *N*_c_.

Next, we allowed for population sizes to change by an order of magnitude between seasons to mimic the seasonal boom-bust cycles observed in *D. melanogaster* populations in temperate climates (i.e. we simulate sizes peaking at 1 million in summer and declining to 100,000 in winter). Rerunning each empirical parameter set with these alternating population sizes resulted in *N*_e_ reductions that scaled with the harmonic mean population size, producing *N*_e_ values an order of magnitude below the census population sizes in the summer period, or proportional decreases similar to the constant size model when scaled against the harmonic mean population size (Fig. 2b).

We also conducted an exploratory analysis of an asymmetric seasonal regime (AR), simulating 3 parameter combinations (*l* = 127, 37, and 18; *y* = 15, 2, and 4.5, respectively) with 8 scaled generations in summer and 2 in winter. Fewer loci remained segregating under the asymmetric regime than under the corresponding symmetrical regime, but this was not consistently associated with weaker reductions in variance *N*_e_ (Supplementary Fig. 10). The relationship between maximum allele-frequency amplitude and the reduction in variance *N*_e_ remained qualitatively similar under the 2 regimes (Supplementary Fig. 11).

Finally, previous theoretical work has shown that the SL model can produce unrealistically large offspring numbers under some scenarios, resulting in high reproductive variance where some individuals contribute unrealistically large offspring numbers to the subsequent generation (Wittmann et al. 2017). Accordingly, we reran our simulations imposing a limit on the maximum number of offspring per parent to 20, observing only a minor reduction in the simulated allele frequency fluctuations (Fig. 2c) that agreed with the subtle change in the allele frequency fluctuations reported in theoretical results after imposing similar offspring caps (Wittmann et al. 2017). Further, this offspring cap had little effect on levels of *N*_e_ reductions when the uncapped reduction was approximately 50% or less, though maximum reductions were limited to approximately 60% for scenarios where the uncapped *N*_e_ reduction was more severe.

## Discussion

In recent years, fluctuating selection has emerged as a novel evolutionary factor that plays an important role in adapting cosmopolitan *Drosophila* populations and other species to their temporally varying habitats (Johnson et al. 2023). These findings have prompted the development of theoretical models to study the allele frequency dynamics of adaptively fluctuating loci and their broader impact on linked neutral variation (Barton 2000; Huerta-Sanchez et al. 2008; Taylor 2013; Wittmann et al. 2017; Wittmann et al. 2023). In this study, we show that fluctuating selection leads to *N*_e_ values that are ∼50% smaller than expected under neutrality when simulating polygenic seasonal selection in an empirically informed *Drosophila* model, with the scale of the decrease being primarily driven by the locus experiencing the largest frequency oscillations among all adaptively fluctuating loci. Our simulations specifically examine the consequences of seasonal alleles already segregating in the population, rather than their complete evolutionary trajectory from de novo mutation to long-term equilibrium. Consequently, the estimated reduction in effective population size reflects the effects of fluctuating selection on standing seasonal variation rather than the complete life cycle of fluctuating alleles. Nonetheless, our results clearly demonstrate the potent ability of fluctuating selection to reduce variance effective population size. This dramatic effect is a direct result of the exaggerated reproductive variance created by individuals repeatedly cycling between high and low fitness as environmental pressures continually alternate (Wittmann et al. 2023), as demonstrated by the greatest *N*_e_ reductions occurring right after the transition to a new season when reproductive variance peaks (Fig. 3). The pronounced reductions in variance effective population size immediately after seasonal transitions may also have biological consequences beyond their contribution to the cycle-averaged estimate. Because the cumulative strength of drift over time is often approximated by the harmonic mean of effective population size across generations, short periods of strongly reduced variance effective size could disproportionately affect allele-frequency dynamics. Such transient bottlenecks may therefore influence the efficacy of selection and the fixation or loss probabilities of weakly selected alleles (Otto and Whitlock 1997). However, the consequences for fixation probabilities are unlikely to be captured fully by a single averaged effective size, because the timing of reduced effective size relative to an allele's frequency trajectory can also be important. Future work should directly examine how these short episodes of reduced variance effective size shape the fate of new and standing variants under fluctuating selection.

There are limitations to our study: our simulation model assumes soft selection, no migration, discrete nonoverlapping generations, an equal number of generations per season, numerous seasonal mutations being maintained, and the specific segregation-lift model of multilocus fluctuating selection by Wittmann et al. It is also important to note that we use seasonal environments as an example of an alternating selective regime and not as a complete representation of seasonality experienced by temperate *Drosophila* populations. Furthermore, our simulations should be interpreted as modeling standing seasonal variation rather than the de novo origin of seasonal alleles. Although all seasonal alleles are introduced at 50% frequency, many are subsequently lost during the simulations, and we compare the final number of segregating loci and their allele-frequency amplitudes to empirical estimates from natural and experimental *Drosophila* populations. This comparison helps ensure that the simulated parameter combinations used for estimating variance effective population size fall within a biologically relevant range. We aimed for our study to provide a baseline understanding of the relationship between fluctuating selection and variance effective population size from which additional biological realism could be added in future. Additionally, future work should investigate models where seasonal alleles arise through mutation and reach their long-term frequency distribution under fluctuating selection prior to estimating their effects on variance effective population size.

Previous work investigating the effect of natural selection on effective population size has also included its consideration as a possible explanation for Lewontin's Paradox (LP), the unexpected decoupling of neutral diversity and *N*_c_ across populations (Lewontin 1974), due to its efficacy also increasing as population size increases (Lewontin 1974; Corbett-Detig et al. 2015; Roberts 2015; Buffalo 2021; Charlesworth and Jensen 2022; Achaz and Schertzer 2023; Kaushik 2023). While fluctuating selection has been proposed as a potential contributor to LP (Buffalo 2021), our simulations show that the resulting reductions in *N*_e_ are largely independent of census size (*N*_c_). This indicates that fluctuating selection alone is insufficient to explain LP. This conclusion is consistent with recent work suggesting that the effects of linked selection, including recurrent selective sweeps, are generally too weak to account for the observed compression of genetic diversity across species (Coop 2016).

Nonetheless, fluctuating selection may still contribute to variation in the ratio of *N*_e_ to *N*_c_ among taxa. In particular, species such as arthropods, which experience strong and recurrent environmental fluctuations on generational timescales, may be especially affected due to the cumulative impact of temporally variable selection on reproductive variance. Unlike models based on recurrent positive selection, which require a sustained input of beneficial mutations and allow diversity to recover between sweeps (Buffalo 2021; Charlesworth and Jensen 2022; Achaz and Schertzer 2023), fluctuating selection can impose persistent reductions in *N*_e_ as long as environmental conditions continue to alternate.

Together, these results suggest that fluctuating selection is unlikely to resolve LP on its own, but may act in concert with other processes, including demography and linked selection, to shape broad-scale patterns of genetic diversity.

### Conclusions and future work

Our findings emphasize that fluctuating selection is likely a fundamental, yet under-appreciated, determinant of effective population size among *Drosophila* and other species prone to temporal environmental shifts. Investigation of fluctuating selection is currently in its infancy, and our understanding of the breadth of fluctuating selection is hindered by the majority of prior empirical research focusing upon *Drosophila* species (Bergland et al. 2014; Machado et al. 2021; Behrman and Schmidt 2022; Rudman et al. 2022; Bitter et al. 2024; Nunez et al. 2024), despite other ectothermic species being obvious candidates for investigation due to their life-histories being highly sensitive to thermal environments (Pfenninger and Foucault 2022; Johnson et al. 2023; Pfenninger et al. 2023; Lynch et al. 2024). Beyond the need for more work on experimental and natural populations from diverse species, additional investigation is required to determine whether the *N*_e_ effects observed in our simulations are robust to different models of fluctuating selection as these emerge in the future (Bertram and Masel 2019). Further, while our analyses show that *N*_e_ reductions are relatively insensitive to changes in population size and restrictions on the number of offspring per parent, future research needs to examine the impact of other evolutionary and demographic forces, including seasonal asymmetries in generation number, continuous changes in population size, and the inclusion of background selection, as well as eco-evolutionary parameterizations tailored toward different species. This will enhance our understanding of how fluctuating selection shapes effective population size and contributes to genome-wide patterns of genetic variation across diverse species.

## Supplementary Material

jkag252_Supplementary_Data

## Data Availability

All simulation code, analysis scripts, and processed data underlying the variance effective population size analyses are available through Zenodo (DOI: 10.5281/zenodo.21960020) and GitHub (https://github.com/olivia-johnson/FluctuatingSelectionNe). Because of their size, the full simulation outputs used to develop the regression model are not archived but can be regenerated using the available simulation code. Empirical seasonal allele frequency data are available from the Dryad repositories associated with Bergland et al. (2015) and Machado et al. (2019). Supplemental material available at G3 online.
