# Supplementary material for: Seasonal fluctuations in fitness result in severe reductions in effective population size
Source: G3 (Bethesda). 2026 Sep 3;16(11):jkag252. doi: 10.1093/g3journal/jkag252 (PMC13732426; doi:10.1093/g3journal/jkag252)
Supplement: Supplementary file 1 [file jkag252_supplementary_data.pdf]

Supplementary Information

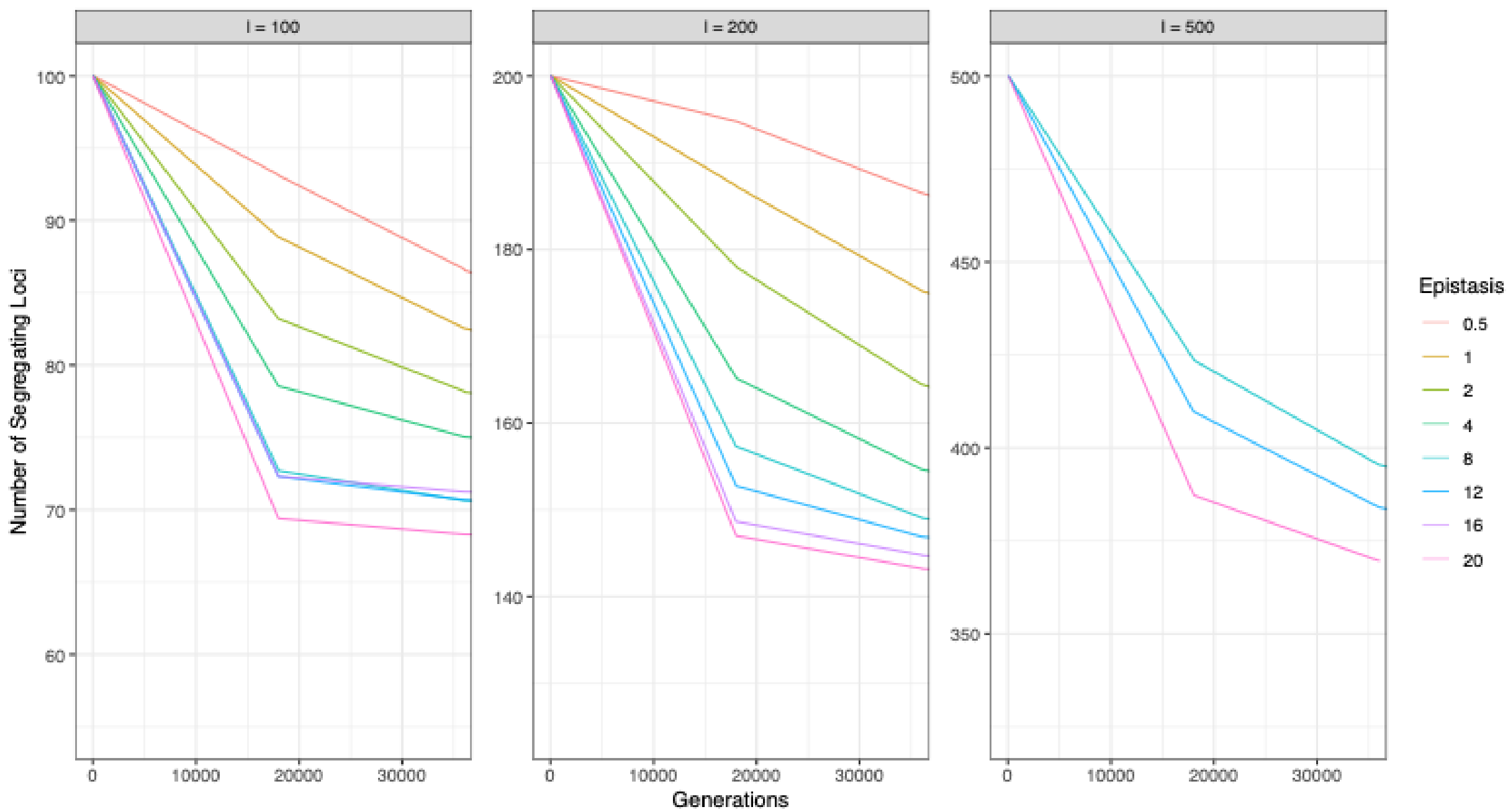

Fig S1. The average number of segregating loci across time.

The time in generations is shown on the x-axis, with the average number of segregating loci shown on the y-axis. Each panel depicts the number of segregating loci for a different number of initial loci. Each line is for a specific epistasis value (*y*), as indicated by the colour of the line.

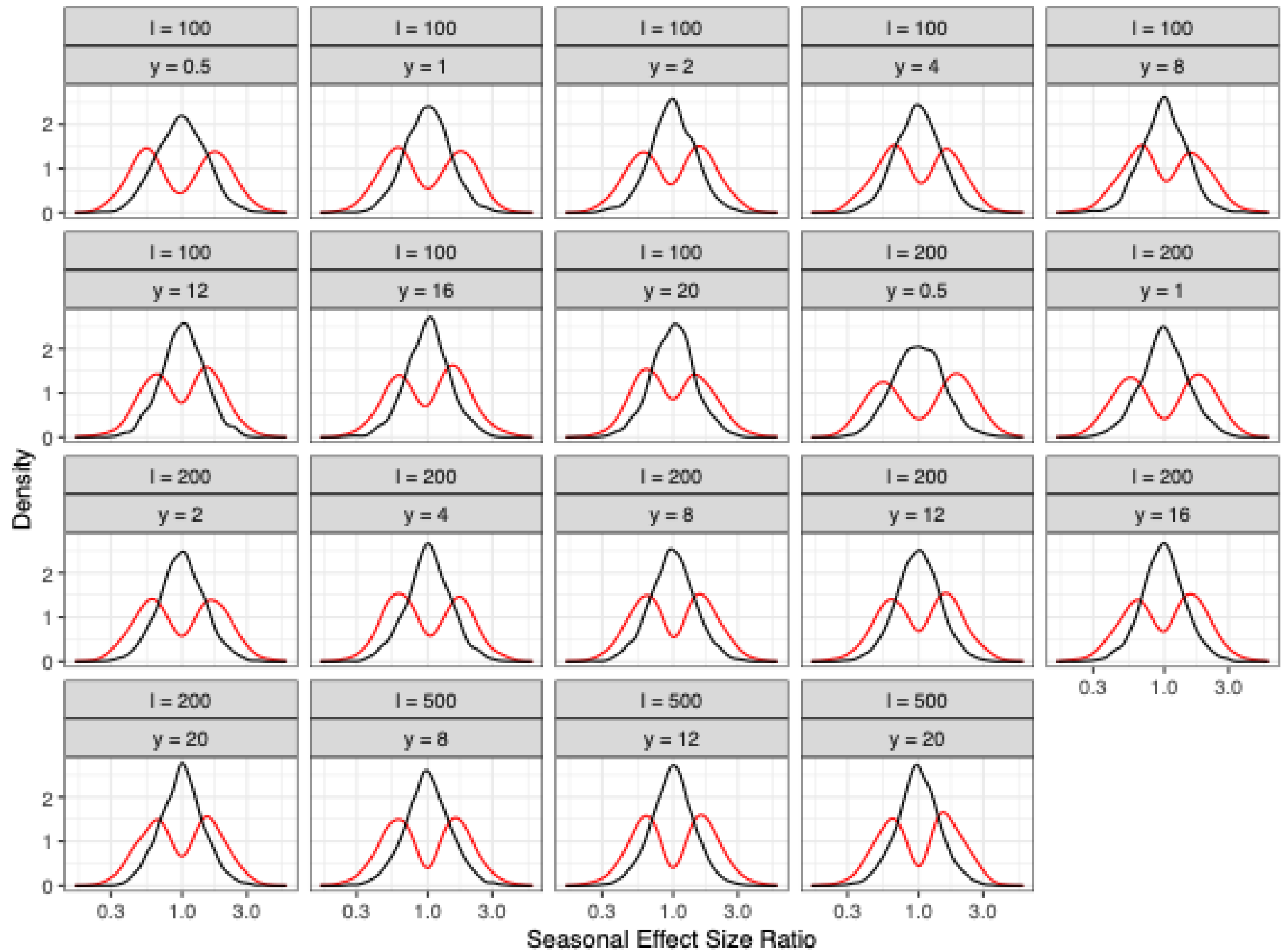

Fig S2. Distribution of the seasonal effect size ratio for segregating (black) and fixed/lost (red) alleles.

The number of initial loci and *y* parameters are labelled at the top of each panel. A seasonal effect size ratio of 1 signifies that the summer and winter effect sizes are equal. The effect size ratio is calculated at the quotient of the summer and winter effect size.

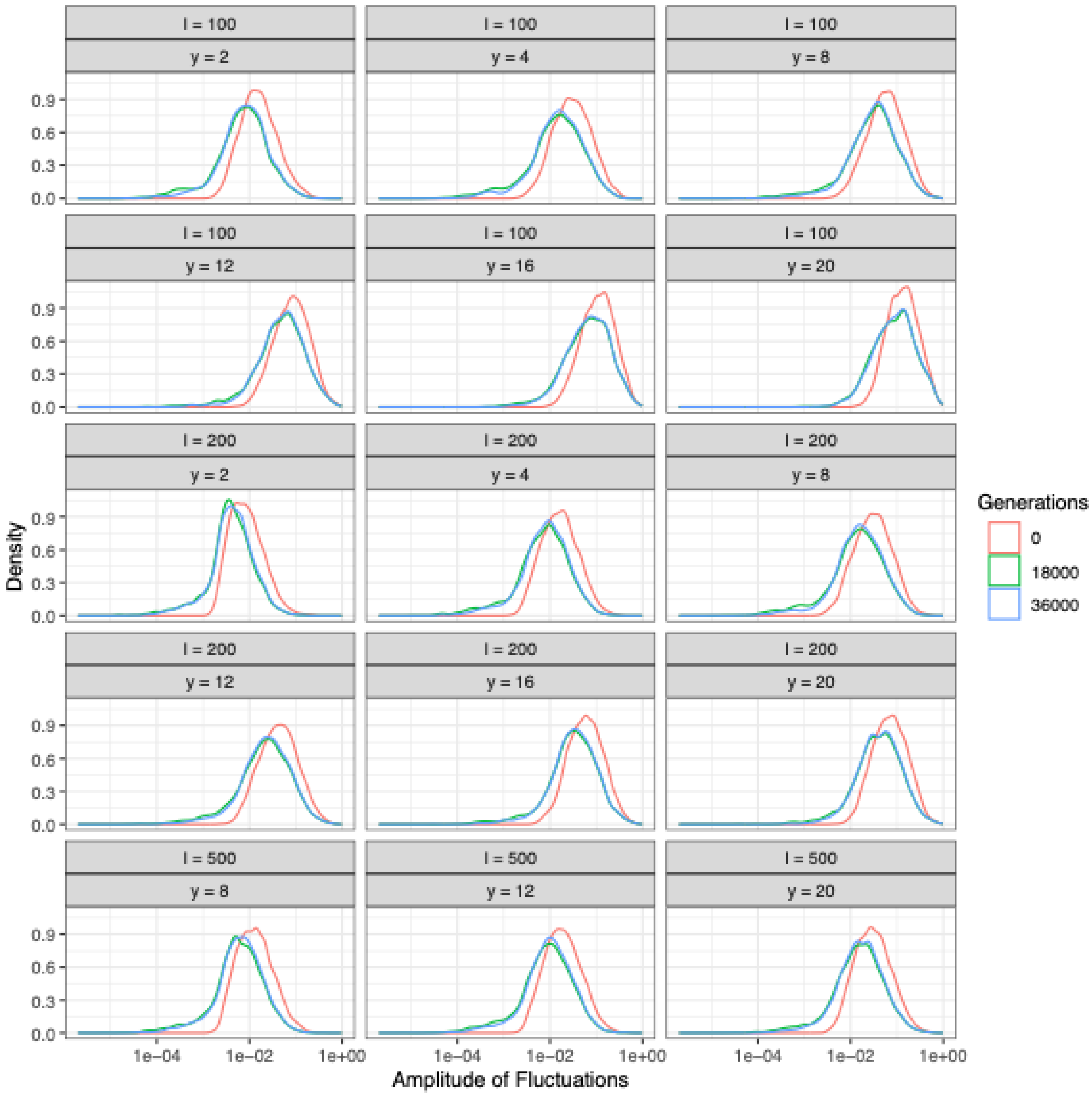

Fig S3. Distribution of the amplitude of allele frequency fluctuations across time.

The distribution of allele frequency fluctuations of segregating seasonal loci at different sampling points (in generations; shown by the colour of the distributions). Each facet displays a different initial loci number (*l*) and epistasis (*y*). The distribution of the amplitude of allele frequency fluctuations is shown on the y-axis on a logarithmic scale. Note that fixed or lost alleles (i.e. non-polymorphic loci) are excluded from the distribution.

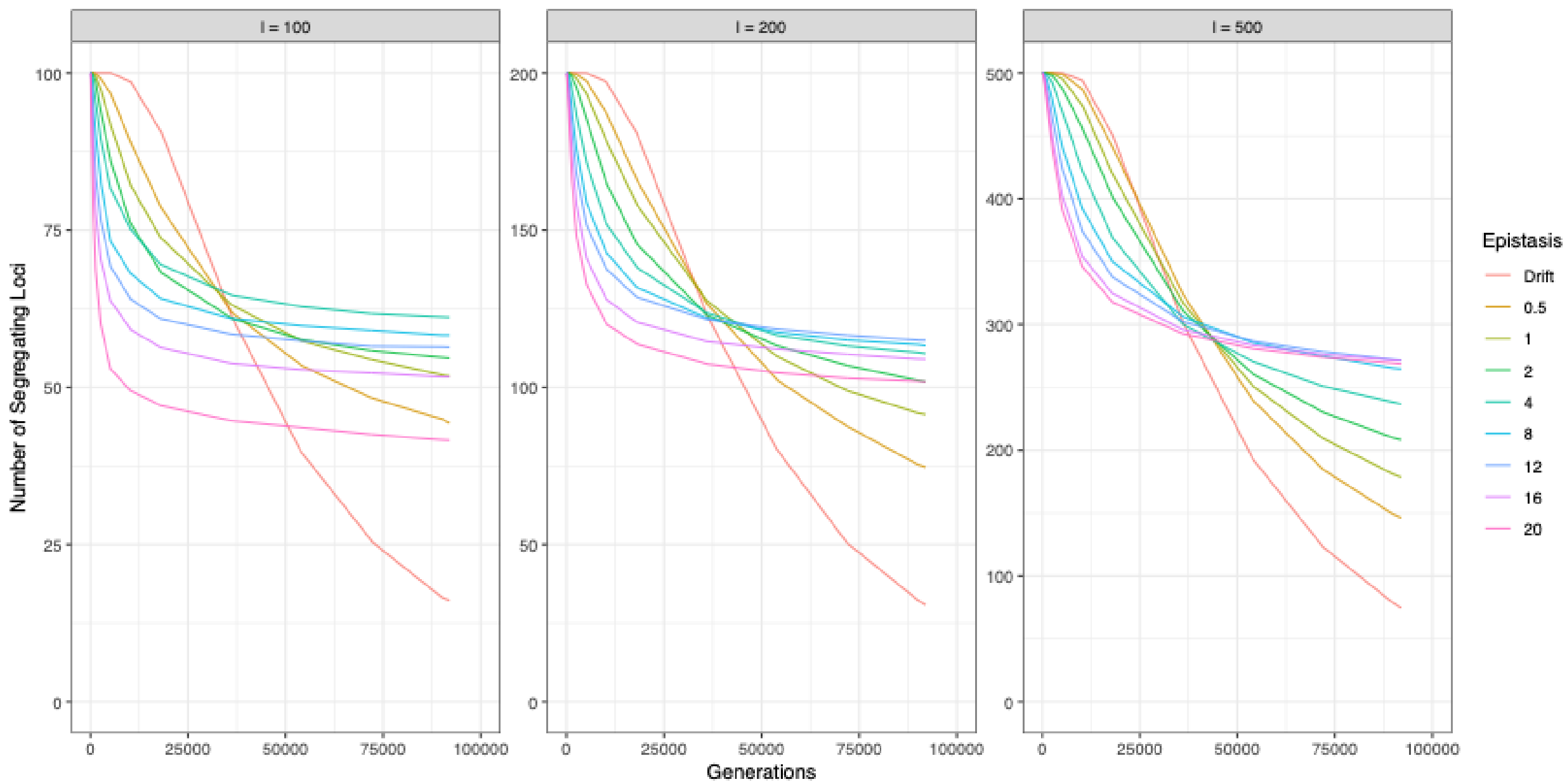

Fig S4. Number of segregating loci across time for simulations with a reduced population size (*N* = 10,000).

We conducted allele frequency simulations that were downscaled by 10 and had increased temporal sampling to improve resolution. We simulated a population of 10,000, with 10 generations per season, and increased sampling in the first 18,000 generations. We observe similar patterns to simulations downscaled by 2, with either a continuous slow decrease in segregating loci for smaller epistasis parameters, or a sharp decrease followed by a stable level of segregating loci. All values of epistasis parameters maintained more loci than under a neutral model (*Drift*). Less segregating loci are maintained in these simulations compared to the original simulations (Figure S1) as the number of generations per season was not further downscaled (i.e. we maintained 5 simulated generations per season rather than downscaling to 1 generation per season for the further downscaled case). This meant seasonal alleles spent more time in each season and were pushed towards fixation or loss more so than alleles in the original simulations. Additionally a smaller population size leads to a greater effect of genetic drift which may have also contributed to this.

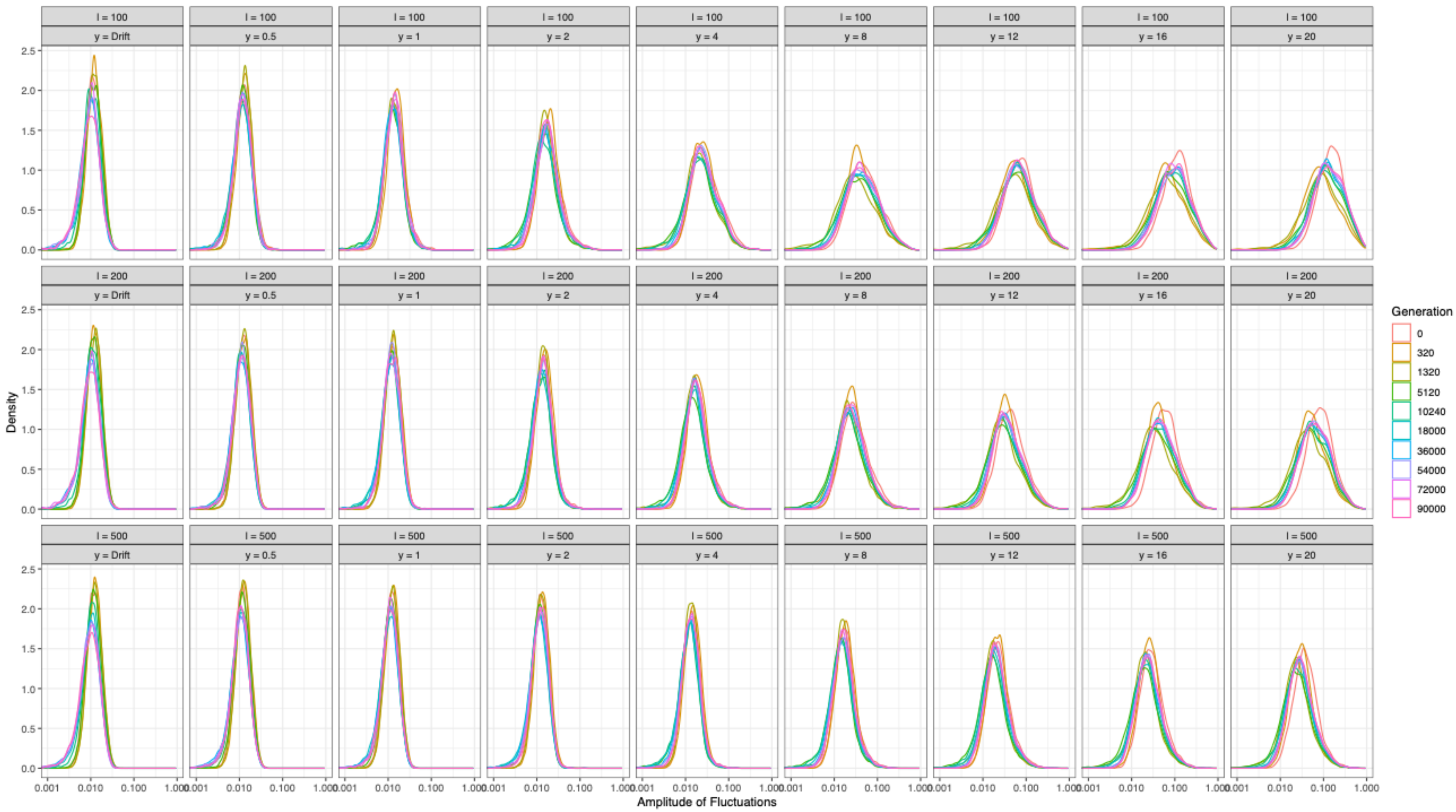

Fig S5. Distribution of allele frequency fluctuations for downscaled simulations.

The distribution of allele frequency fluctuations for simulations with 10,000 individuals and increased temporal sampling. For cases of seasonally fluctuating selection, the distribution is generally stable by 1,200 simulated generations.

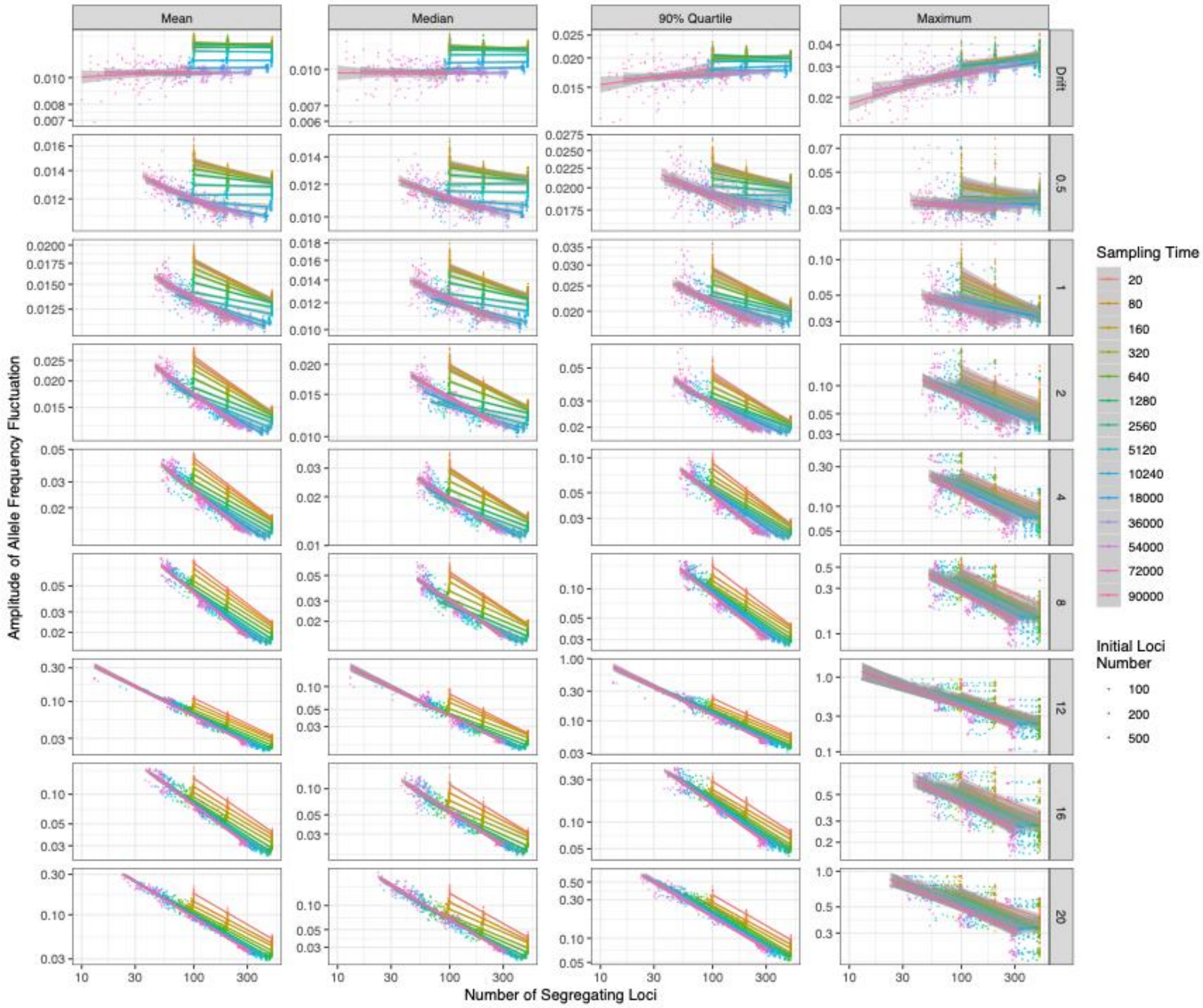

Fig S6. Relationship between amplitude of allele frequency fluctuation and number of segregation loci over time.

The relationship between amplitude of allele frequency fluctuation and number of segregating loci over time (both on a logarithmic scale) for simulations with 10,000 individuals and increased temporal sampling. The linear relationship between the amplitude and loci number converges over time, with faster convergence for greater epistasis values. Sampling time is given as the first generation of the sampling period (the following 3 consecutive seasonal cycles).

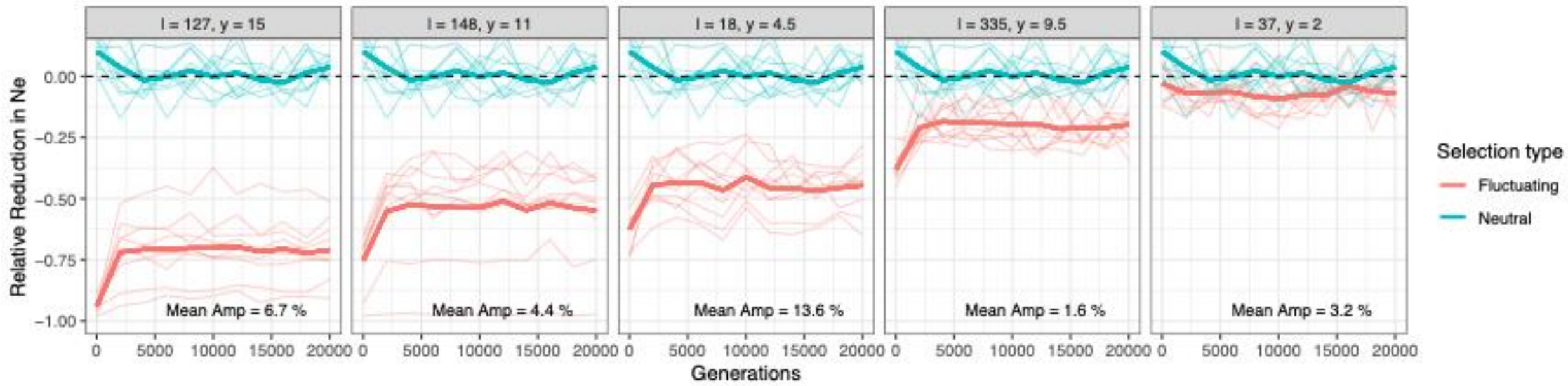

Fig S7. The relative reduction in N_e with replicate values.

The relative reduction in effective population size across time. Each panel shows effective population size for a different combination of initial loci number and epistasis value (labelled respectively in the top strip). A dashed black line depicts the neutral expectation (i.e. 0% reduction). The bold blue line demonstrates relative effective population size under neutral evolution with thin blue lines showing the values for single replicates, while the red lines show the reduction in effective population size under fluctuating selection. The corresponding mean amplitude of each parameter combination is included in the bottom right of each panel.

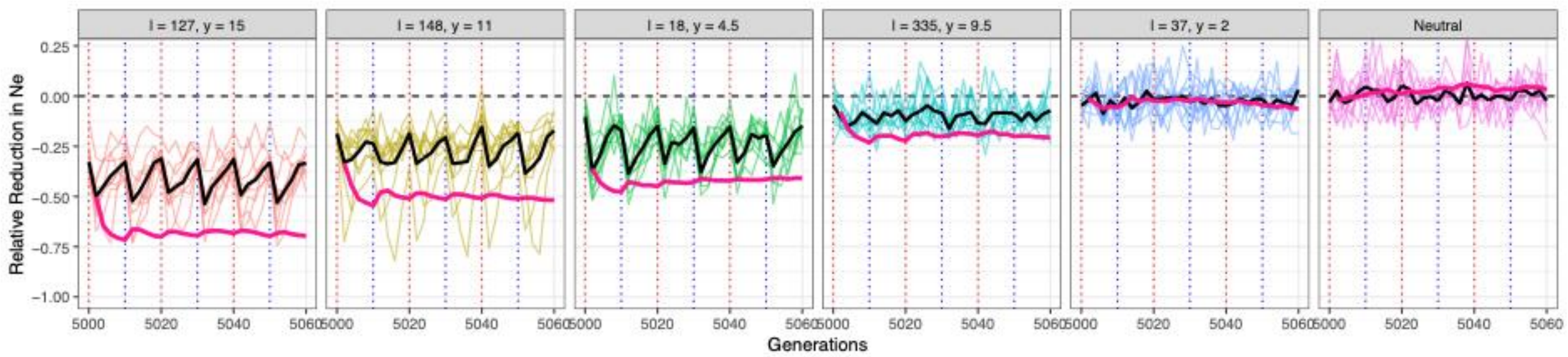

Fig S8. Variance in effective population size over the seasonal cycle for three consecutive years.

Effective population size for different fluctuating selection parameters (coloured lines; labelled at the top of each panel) across the seasonal cycle, 2 seasons each consisting of 10 generations. The thin lines show the values for each replicate while the thick black line is the average of all 10 replicates. The red and blue dashed vertical lines signifies the start of the summer and winter seasons, respectively. N_e was sampled from generation 5001 to 5060 of the simulation as previous simulations suggested effective population size reduction had become stable at this time. We also calculated variance N_e over cumulative numbers of generations (2 to 60 [1:30 simulated generations]; bold pink line) to visualise the effect of increasing the number of generations over which the variance in allele frequency is captured.

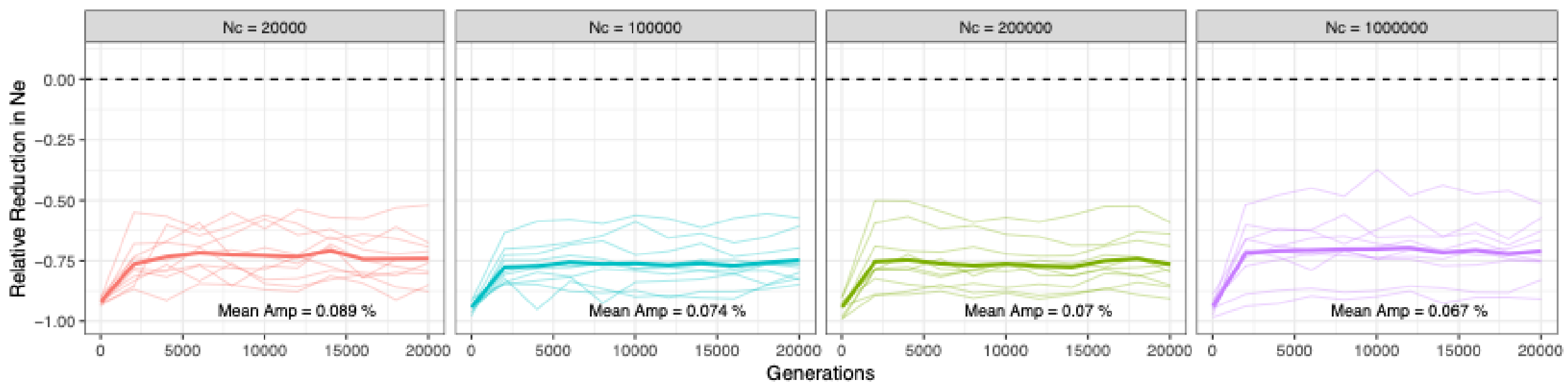

Fig S9. Reduction in effective population size for varying census size (N_c).

Reduction in N_e for four constant population sizes, 20000, 100000, 200000, and 1000000, labelled in the top strip of each panel. Replicate values are shown by the thin coloured lines, while the mean is shown in the bold coloured line. The mean amplitude of seasonal loci is shown at the bottom of the panel. The black dashed line represents the census population size.

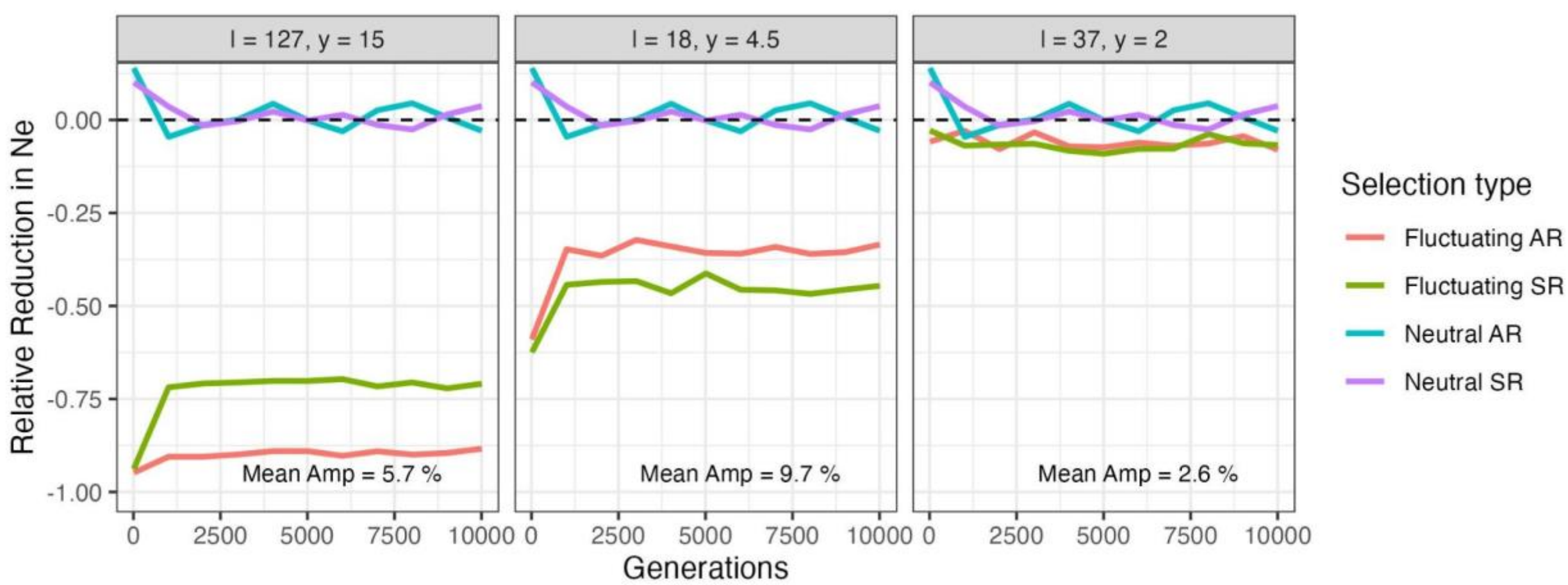

Fig S10. Variance effective size under an asymmetrical seasonal regime.

Variance effective population size under fluctuating selection and neutrality when under a symmetrical seasonal regime (SR) and an asymmetrical seasonal regime (AR). Mean amplitude of loci under Fluctuating AR is provided in each panel, respective to the simulation conditions displayed at the top. Each condition was averaged over 10 simulated replicates.

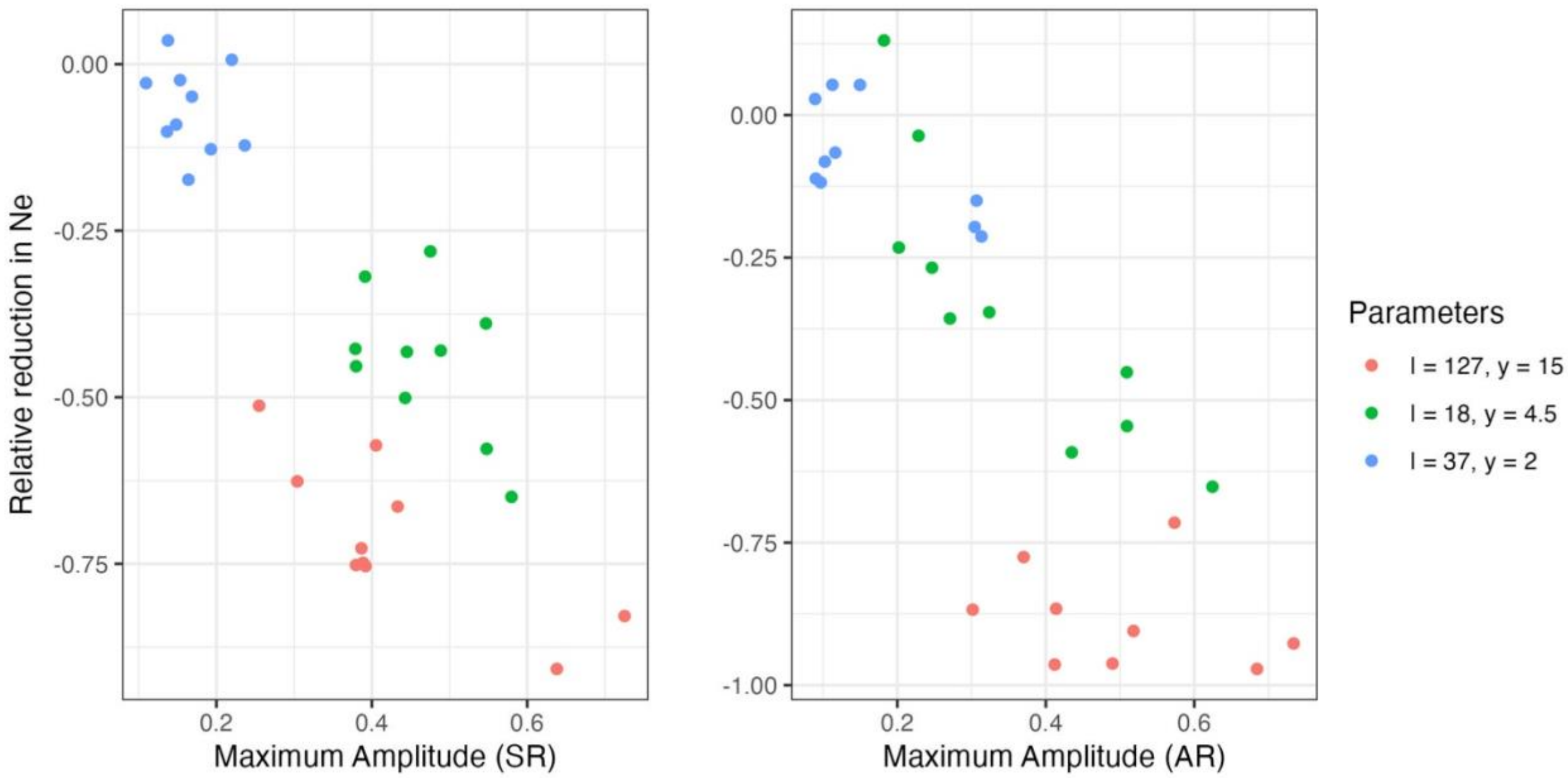

Fig S11. Maximum amplitude under an asymmetrical seasonal regime.

Relationship between maximum amplitude of fluctuating loci under symmetrical (SR) and asymmetrical (AR) seasonal regimes and the relative reduction in effective population size. Each condition was simulated 10 times, with each replicate a point on the plot.

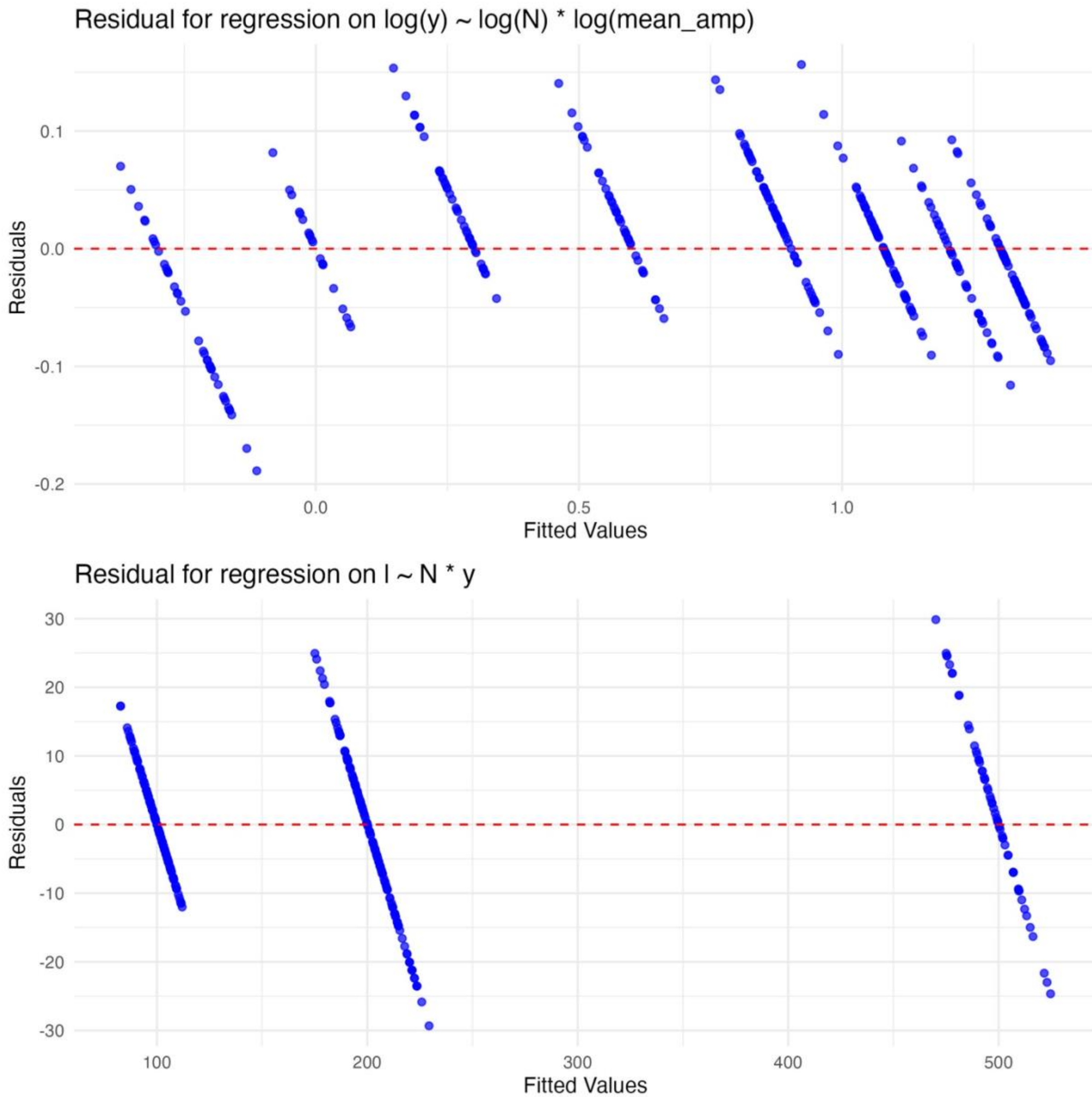

Fig S12. Residual plot for regression models.

The top panel illustrates the residuals for equation 3, predicting the epistasis value. The bottom panel depicts the residuals for equation 4, which predicts the number of initial loci to introduce to the simulation.

Table S1. Evaluating regression approach and derived parameters and comparing simulations results to empirical values.

Empirical Final Loci Number	Derived Initial Loci Number	Empirical Mean Amplitude	Mean Amplitude of All Replicates	95% Confidence Interval Final Loci Number	95% Confidence Interval Mean Amplitude
111	148	0.05	0.0484	110-115	0.0458-0.0510
90	127	0.08	0.0741	88-97	0.0697-0.0784
264	336	0.02	0.0180	260-268	0.0169-0.0190
27	37	0.04	0.035	25-29	0.0311-0.0388
9	18	0.35	0.150	13-16	0.0139-0.0161

Table S2. Model fit information for multiple linear regression models.

Value	Coefficient Estimate	Standard Error	Equation
**Intercept**	-0.15320	0.11546	[3]
**Log(n)**	1.30964	0.05679	[3]
**Log x)**	0.76593	0.07027	[3]
**Log(n)*log(x)**	0.18049	0.03385	[3]
**Intercept**	4.4069745	1.9938219	[4]
**n**	1.1537026	0.0121766	[4]
**y**	0.4430562	0.1609967	[4]
**n*y**	0.0090322	0.0009159	[4]
